# P-847. Duration of Daptomycin and Ceftaroline Dual Therapy in Salvage Methicillin Resistant *Staphylococcus aureus* (MRSA) Bacteremia Prior to Monotherapy De-escalation

**DOI:** 10.1093/ofid/ofae631.1039

**Published:** 2025-01-29

**Authors:** Josh Lechner, Patrick M Kinn, Dilek Ince, Kelly M Percival

**Affiliations:** Nebraska Medicine, Iowa City, Iowa; University of Iowa Hospitals & Clinics, Iowa City, Iowa; University of Iowa Hospitals & Clinics, Iowa City, Iowa; University of Iowa Health Care, Iowa City, Iowa

## Abstract

**Background:**

Methicillin resistant *Staphylococcus aureus* (MRSA) bacteremia is a serious medical condition known to cause mortality. Earlier escalation to daptomycin and ceftaroline is associated with improved outcomes compared to monotherapy; however, the optimal duration of dual therapy after bloodstream clearance remains unclear.

**Figure:**
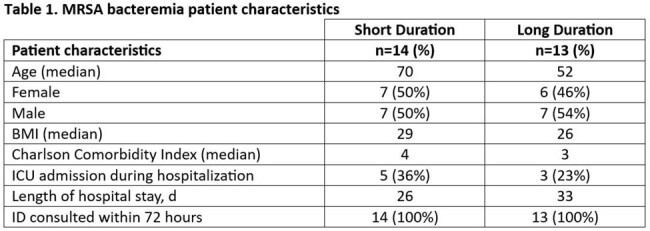

**Methods:**

This was a single center retrospective cohort study comparing a composite outcome of clinical failure (90-day mortality, 90-day readmission, and bacteremia recurrence) in adult patients with MRSA bacteremia between June 2018 and June 2023 who received dual therapy. Patients who were treated with combination daptomycin and ceftaroline were divided into two groups: patients treated with dual therapy for less than or equal to ten days (short) and patients treated with dual therapy for greater than ten days (long).

**Figure:**
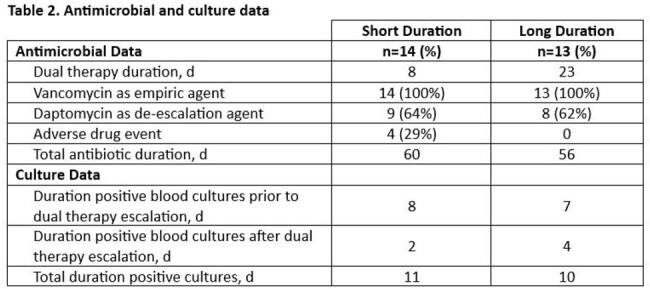

**Results:**

The study population included 14 patients in the short group and 13 patients in the long group with 48% female (n=13), 96% white (n=26), median age of 62 years (IQR 54-70), with additional demographics shown in Table 1. There was no statistically significant difference in the primary composite clinical failure outcome (50% vs 38%, p=0.70) which included 90-day infection-related mortality (36% short vs 23% long, p=0.67), 90-day readmission (29% short vs 23% long, p=1), and bacteremia recurrence (7% short vs 0% long). Culture data and antimicrobial usage is shown in Table 2, with patients in the long group receiving a mean of 15 days more of dual therapy than the short group. Patients in the long dual therapy group had longer duration of positive blood cultures after escalation to daptomycin and ceftaroline when compared to the short group (4 days long vs 2 days short) and had longer median hospital length of stays (22 days short vs 25 day long).

**Conclusion:**

Among patients with persistent MRSA bacteremia, short durations of dual therapy with ceftaroline and daptomycin may be adequate once bloodstream clearance is achieved. This study found no difference in 90-day mortality, recurrence, or readmission among patients who received short vs long durations of dual therapy. Further research is needed to identify the optimal duration of dual therapy.

**Disclosures:**

**All Authors**: No reported disclosures

